# Venous Tumor Thrombus Across Malignancies: Imaging, Management, and Clinical Implications

**DOI:** 10.3390/cancers18213422

**Published:** 2026-10-22

**Authors:** Haijie Liang, Jingjun Jiang, Songyi Guo, Xu Tang, Xingyu Liu, Feiyang Qi, Guyue Bian, Kuisheng Liu, Siyi Huang, Boyang Wang, Shidong Wang, Jun Wang, Taiqiang Yan, Yunfan Wang, Kunkun Sun, Wei Guo, Jichuan Wang, Yi Yang

**Affiliations:** 1 Beijing Key Laboratory for Musculoskeletal Tumors, Musculoskeletal Tumor Center, Peking University People’s Hospital, Beijing 100044, China2210122506@stu.pku.edu.cn (G.B.);; 2 Department of Vascular Surgery, Peking University People’s Hospital, Beijing 100044, China; 3 Department of Hepatobiliary Surgery, Peking University People’s Hospital, Beijing 100044, China; 4 Department of Urology, Peking University People’s Hospital, Beijing 100044, China; 5 Department of Orthopedics, Peking University First Hospital, Beijing 100034, China; 6 Department of Pathology, Peking University Shougang Hospital, Beijing 100144, China; 7 Department of Pathology, Peking University People’s Hospital, Beijing 100044, China

**Keywords:** venous tumor thrombus, renal cell carcinoma, hepatocellular carcinoma, sarcoma, imaging, surgical management, multidisciplinary treatment, prognosis

## Abstract

Venous tumor thrombus (VTT) is a clinically important manifestation of malignancy that creates diagnostic, therapeutic, and prognostic challenges. Evidence is highly uneven across tumor types: renal cell carcinoma and hepatocellular carcinoma account for much of the available clinical experience, whereas data for rarer tumors, including sarcomas, remain limited. This narrative review was based on a structured PubMed search supplemented by relevant publications identified through reference checking and subsequent topic-specific reading. We review the incidence, imaging evaluation, surgical and non-surgical management, and prognosis of VTT across major malignancies. Although several considerations are shared across tumor types, including assessment of thrombus extent, venous wall involvement, venous patency, collateral circulation, resectability, and systemic disease status, their clinical implications differ according to the underlying malignancy. In particular, treatment strategies established in well-studied tumors should not be directly extrapolated to rarer malignancies with different tumor biology, treatment sensitivity, and patterns of disease progression. A cross-tumor approach may therefore be most useful for defining common questions in VTT assessment and multidisciplinary planning, while treatment decisions remain tumor-specific. Future progress will require standardized reporting, multicenter collaboration, and better integration of emerging systemic therapies into multidisciplinary care.

## Simple Summary

Venous tumor thrombus (VTT) occurs when malignant tumor tissue extends macroscopically into the venous system and can substantially affect diagnosis, treatment planning, surgical complexity, and prognosis. Clinical experience is greatest in renal cell carcinoma and hepatocellular carcinoma, whereas evidence for rarer malignancies, including sarcomas, remains limited. This narrative review summarizes the incidence, imaging evaluation, surgical and non-surgical management, and prognostic implications of VTT across major malignancies. Particular attention is given to distinguishing VTT from bland thrombus and primary venous malignancies and to evaluating thrombus extent, venous wall involvement, venous patency, and collateral circulation. Although these assessment principles provide a useful framework across tumor types, treatment decisions cannot be directly extrapolated between malignancies because tumor biology, treatment sensitivity, and patterns of disease progression differ substantially. Multidisciplinary, tumor-specific decision-making and more standardized multicenter research are needed to improve the management of VTT.

## 1. Introduction

Venous tumor thrombus (VTT) is a distinctive manifestation of malignancy characterized by macroscopic intravascular extension of tumor tissue within the venous system [1,2,3,4,5,6]. VTT usually arises through direct extension of tumor into adjacent veins and may subsequently extend into larger venous structures. It should be distinguished from microscopic vascular invasion, discontinuous tumor embolism, and bland thrombus, which differ in their clinical implications and management. Primary venous malignancies are also distinct from VTT and are considered mainly in the differential diagnosis of an intravascular mass. The presence of VTT may increase surgical complexity and, depending on the underlying malignancy, may also be associated with advanced disease and adverse prognosis [6,7,8]. 

Current evidence regarding VTT is highly uneven. Substantial clinical experience has accumulated in renal cell carcinoma (RCC) and hepatocellular carcinoma (HCC), in which VTT is relatively common, well recognized, and extensively studied [1,2,4,5,9,10,11]. These tumor types have provided important insights into imaging evaluation, anatomical classification, operative planning, perioperative management, and prognostic assessment. In contrast, for rarer VTT-associated malignancies, including osteosarcoma and other sarcomas, available evidence remains sparse, fragmented, and often limited to small retrospective series or case reports [3,6,12]. This imbalance makes it difficult to establish tumor-specific management strategies for uncommon clinical scenarios.

Cross-tumor comparison may nevertheless be useful, provided that similarities in vascular anatomy are not assumed to imply similar tumor biology or treatment response. The most transferable principles concern the clinical questions that need to be addressed, including confirmation of VTT, definition of its anatomical extent, assessment of venous wall involvement and collateral circulation, technical resectability, and consideration of systemic disease status. In contrast, the indication for surgery, the role of systemic or locoregional therapy, and the prognostic significance of VTT remain strongly dependent on the underlying malignancy.

This review summarizes the available clinical literature on VTT across major malignancies, with particular emphasis on the more extensive evidence from RCC and HCC. The evidence is organized around incidence, imaging evaluation, surgical management, non-surgical treatment, and prognosis, followed by a discussion of principles that may be shared across malignancies and those that should remain tumor-specific. The major clinical characteristics of VTT across tumor types are summarized in Table 1.

## 2. Literature Search and Scope

This narrative review was based on a structured literature search of PubMed. PubMed was searched using the term ‘tumor thrombus [Title/Abstract]’ for publications from 1 January 1980 to 1 January 2026. The updated search identified 3763 records, which were screened by title and abstract for relevance to the clinical topics addressed in this review. Relevant publications were selected for full-text evaluation. Additional directly relevant studies identified through reference checking and subsequent topic-specific reading were also considered during manuscript preparation and revision. Because the purpose of this review was to synthesize clinically relevant concepts across different malignancies rather than to perform a systematic review or meta-analysis, formal systematic-review eligibility criteria were not applied.

The review focuses on macroscopic venous tumor thrombus associated with malignant tumors. Microscopic vascular invasion and discontinuous tumor emboli were not considered VTT for the purpose of this review, although they are discussed where relevant to the clinical context. Primary venous malignancies, such as leiomyosarcoma of the inferior vena cava, were likewise not classified as VTT and are discussed only as differential diagnoses. The literature was organized around the main clinical topics addressed in this review: incidence, imaging evaluation, surgical management, non-surgical treatment, prognosis, and cross-tumor considerations for management.

## 3. Incidence of Venous Tumor Thrombus

The incidence of VTT varies substantially according to tumor type, anatomical location, and disease stage [1,2,83,84]. Among solid tumors, VTT is most frequently encountered in RCC and HCC, in which tumor growth patterns and vascular anatomy predispose to intravascular extension. In RCC, venous extension involving the renal vein and/or inferior vena cava (IVC) has been reported in more than 20% of cases, whereas extension specifically into the IVC occurs in approximately 4–10% of patients [1,2,13,14,15,16]. In HCC, VTT most commonly manifests as portal vein tumor thrombus (PVTT). Reported rates of PVTT vary widely, ranging from approximately 8.9% to 46% across different clinical series, reflecting differences in disease stage, patient selection, and diagnostic criteria [11,49,50]. Hepatic vein tumor thrombus (HVTT) and inferior vena cava tumor thrombus (IVCTT) are less frequent, occurring in approximately 1.4% to 4.9% of cases [5,9]. PVTT may coexist in up to 40% of patients with HVTT or IVCTT, usually reflecting more advanced vascular involvement [4,5,10,51,59,106].

In other epithelial malignancies, VTT is less common. Wilms tumor may present with IVCTT or atrial tumor thrombus in approximately 6% to 11.3% of cases [80,81,82]. Testicular tumors are associated with IVCTT in about 0.6% to 2.5% of patients [85,86,87]. VTT has been reported in approximately 1–2% of colorectal cancer cases [88], whereas PVTT in gastric cancer is rare but associated with poor prognosis [89]. Although microscopic vascular invasion is relatively common in some thyroid malignancies, macroscopic VTT is rare [83,84].

Mesenchymal tumors, including sarcomas, can also present with VTT, although available data are limited and the reported incidence varies by histologic subtype [3,6,105,107,108,109,110,111,112]. In a single-institution series of pelvic bone sarcomas, macroscopic VTT was identified in 22.6% of osteosarcomas, compared with 3.8% of chondrosarcomas, 1.4% of Ewing sarcomas, and 3.8% of undifferentiated pleomorphic sarcomas of bone [6]. These observations suggest that both tumor type and anatomical location may influence the likelihood of VTT formation. However, because these estimates were derived from a single-institution pelvic bone sarcoma cohort, they may be influenced by referral and selection bias and should not be interpreted as population-level incidence estimates. Independent validation is lacking.

Overall, reported incidence rates should be interpreted with caution because most data are derived from retrospective series and may be affected by patient selection, disease stage, imaging modality, and diagnostic criteria. These limitations are particularly important when comparing incidence across tumor types.

## 4. Imaging Evaluation of Venous Tumor Thrombus

**Figure 1:**
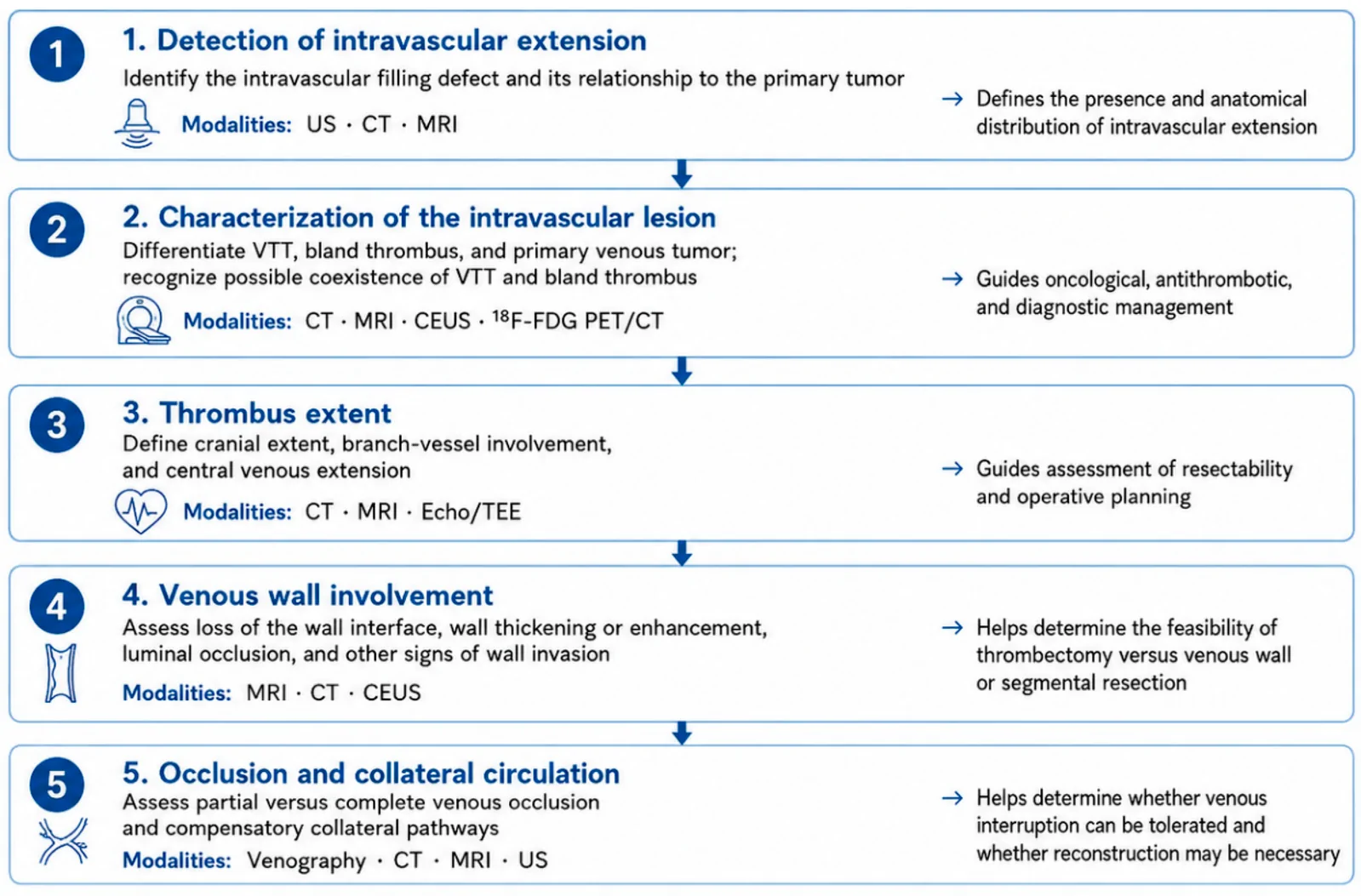
Stepwise imaging evaluation of venous tumor thrombus (VTT). Imaging assessment is organized around five clinical questions: detection of intravascular extension, characterization of the intravascular lesion, definition of thrombus extent, assessment of venous wall involvement, and evaluation of venous occlusion and collateral circulation. The modalities shown represent commonly used imaging options; modality selection depends on the primary tumor, venous territory, clinical question, and local expertise. Abbreviations: VTT, venous tumor thrombus; US, ultrasonography; CT, computed tomography; MRI, magnetic resonance imaging; CEUS, contrast-enhanced ultrasound; PET/CT, positron emission tomography/computed tomography; FDG, fluorodeoxyglucose; TEE, transesophageal echocardiography.

Accurate imaging evaluation is fundamental to the diagnosis and management of VTT because it directly influences treatment planning, surgical strategy, and prognostic assessment [6,7,8,10]. Although imaging protocols vary according to tumor type and venous territory, evaluation can be organized around several common clinical questions, including detection of intravascular tumor extension, differentiation from bland thrombus or other intravascular masses, definition of thrombus extent, assessment of venous wall involvement, and evaluation of venous occlusion and collateral circulation (Figure 1).

The first step is to identify intravascular extension and define its relationship to the primary tumor. In many malignancies, VTT extends from the primary tumor into adjacent veins and subsequently into larger venous structures [3,50,77,113]. HCC-associated PVTT represents a distinct anatomical setting because the portal vein is an afferent vascular system of the liver. HCC may directly invade adjacent portal venous branches and extend within the portal venous tree. Color Doppler ultrasonography can reveal intraluminal filling defects, whereas contrast-enhanced computed tomography (CT) and magnetic resonance imaging (MRI) provide more comprehensive assessment of the anatomical distribution of VTT [114,115,116]. The choice of imaging modality depends on the primary tumor, venous territory, and clinical question. Plain CT has a limited role in characterization of VTT but may demonstrate calcification or ossification within the thrombus, as can occur in osteosarcoma-associated VTT [3,6].

A critical diagnostic consideration is differentiation of VTT from bland thrombus. This distinction has direct implications for clinical management. VTT reflects intravascular tumor extension, whereas bland thrombus represents conventional thrombosis and may require a different therapeutic approach. D-dimer levels have limited diagnostic value because they may be normal or only mildly elevated in chronic bland thrombus and may also be elevated in VTT because of intratumoral hemorrhage [3,6]. Imaging features suggestive of VTT include luminal expansion, enhancement within the thrombus, and MRI signal characteristics similar to those of the primary tumor [3,6,116,117]. In contrast, bland thrombus usually lacks internal enhancement and may show temporal changes in MRI signal [118]. These findings are not universally diagnostic, however, and their value varies according to tumor type, venous territory, and imaging protocol. VTT and bland thrombus may also coexist within the same venous segment. The main imaging features used in this differential diagnosis are summarized in Table 2.

Contrast-enhanced ultrasound (CEUS) may provide additional information in selected settings. In one retrospective series of 60 patients with RCC-associated IVCTT undergoing robot-assisted thrombectomy, intraoperative CEUS differentiated VTT from bland thrombus with reported sensitivity and specificity of 100% and 96%, respectively [128]. These values represent study-specific estimates from a selected RCC population and should not be interpreted as general diagnostic-performance measures for CEUS across different malignancies or venous territories. ^18^F-FDG PET/CT may also assist in differentiation when the underlying malignancy is FDG-avid [123,124,125,126]; however, its diagnostic value depends on the histological type and metabolic activity of the primary tumor, and absence of increased FDG uptake does not exclude VTT.

In HCC, PVTT may demonstrate enhancement during the arterial phase followed by relative washout on portal venous or later phases, although this pattern is not present in every case. Continuity between the thrombus and the primary hepatic tumor, enhancement within the thrombus, and expansion of the involved portal vein further support PVTT rather than bland portal vein thrombosis. Gadoxetic acid-enhanced MRI has also been reported to depict PVTT enhancement and continuity with the primary tumor more frequently than contrast-enhanced CT in selected patients with HCC [127].

Primary venous sarcoma, most commonly leiomyosarcoma of the IVC, should be distinguished from VTT because it arises from the venous wall rather than representing intravascular extension of an extravenous primary tumor [119,120]. Because it is itself a malignant tumor, it shares the imaging hallmarks that separate VTT from bland thrombus, including heterogeneous internal enhancement, diffusion restriction on diffusion-weighted MRI, and increased metabolic activity on ^18^F-FDG PET/CT [120,121,122]. A wall-centered mass, particularly with eccentric or extraluminal growth and no identifiable organ-based primary tumor, favors a primary venous malignancy. This distinction is clinically important because its surgical management differs from that of secondary VTT [119,120,121]. The major imaging differences among VTT, bland thrombus, and primary venous sarcoma are summarized in Table 2.

When imaging remains inconclusive and histological confirmation would alter management, transvenous biopsy may be considered in selected patients with an indeterminate intravascular mass. In a retrospective series of 19 patients with filling defects in the great vessels, endovascular biopsy was technically successful and provided a histological diagnosis in all patients, with no procedural complications reported [129]. Femoral and jugular venous approaches have also been described for biopsy of selected IVC lesions. However, published experience remains limited, and biopsy is generally unnecessary when the diagnosis of VTT is clear from the primary tumor and imaging findings.

Precise assessment of VTT extent is essential because it directly affects treatment planning, surgical approach, and resectability [6,7,8,10]. Ultrasonography can accurately assess thrombus in major veins, but it is less effective for evaluating branch-vessel involvement and may provide information that is less easily shared within a multidisciplinary surgical team [115,134]. In contrast, contrast-enhanced CT and MRI provide comprehensive anatomical information, allowing clinicians to determine the origin, location, cranial extent, and branch-vein involvement of VTT [6,7,8,10,114,117,134]. In selected cases, transesophageal echocardiography (TEE) can be used for intraoperative localization and real-time monitoring of high-level inferior vena cava or superior vena cava tumor thrombus [114,135].

Evaluation of venous wall invasion is another important component of preoperative assessment. In RCC with VTT, reported rates of venous wall invasion vary widely from 22.5% to 70.9% across surgical series [32,136,137,138]. This variation likely reflects heterogeneity in patient selection, thrombus extent, definitions of wall invasion, and operative or pathological assessment. Wall invasion appears less frequently reported in HCC with PVTT [52,53]. When the venous wall is invaded, complete separation of the thrombus from the intima may not be possible, and partial or total venous wall resection may be required [16,137,139,140,141]. Failure to recognize wall invasion may increase the risk of residual tumor and local recurrence [137,142]. MRI has been studied for preoperative assessment of wall invasion mainly in RCC-associated IVCTT. Small retrospective series using operative findings as the reference standard have reported variable diagnostic performance [143,144,145], and these findings should not be directly extrapolated to other malignancies or venous territories. Imaging findings suggestive of wall invasion include altered signal or disruption of the venous wall, wall thickening and enhancement, luminal dilation, complete luminal obstruction, perivascular edema, and irregular thrombus morphology [143,144,145,146]. CEUS has also been investigated for assessment of IVC wall invasion in RCC [128].

Finally, assessment of collateral circulation is important when venous resection or ligation is anticipated. Because VTT-related venous occlusion usually develops gradually, compensatory collateral pathways can form and reduce venous return pressure [1,33,141,146]. The adequacy of collateral circulation influences whether venous interruption can be tolerated and whether reconstruction may be necessary [114,146,147]. Venography provides direct assessment of collateral pathways, while CT, MRI, and ultrasonography can also provide useful information when interpreted together with clinical signs [134,141]. Complete chronic venous occlusion with well-developed collateral circulation and no evidence of venous return disorder may indicate that venous ligation or resection without reconstruction can be tolerated in selected patients [141].

## 5. Surgical Management of Venous Tumor Thrombus

**Figure 2:**
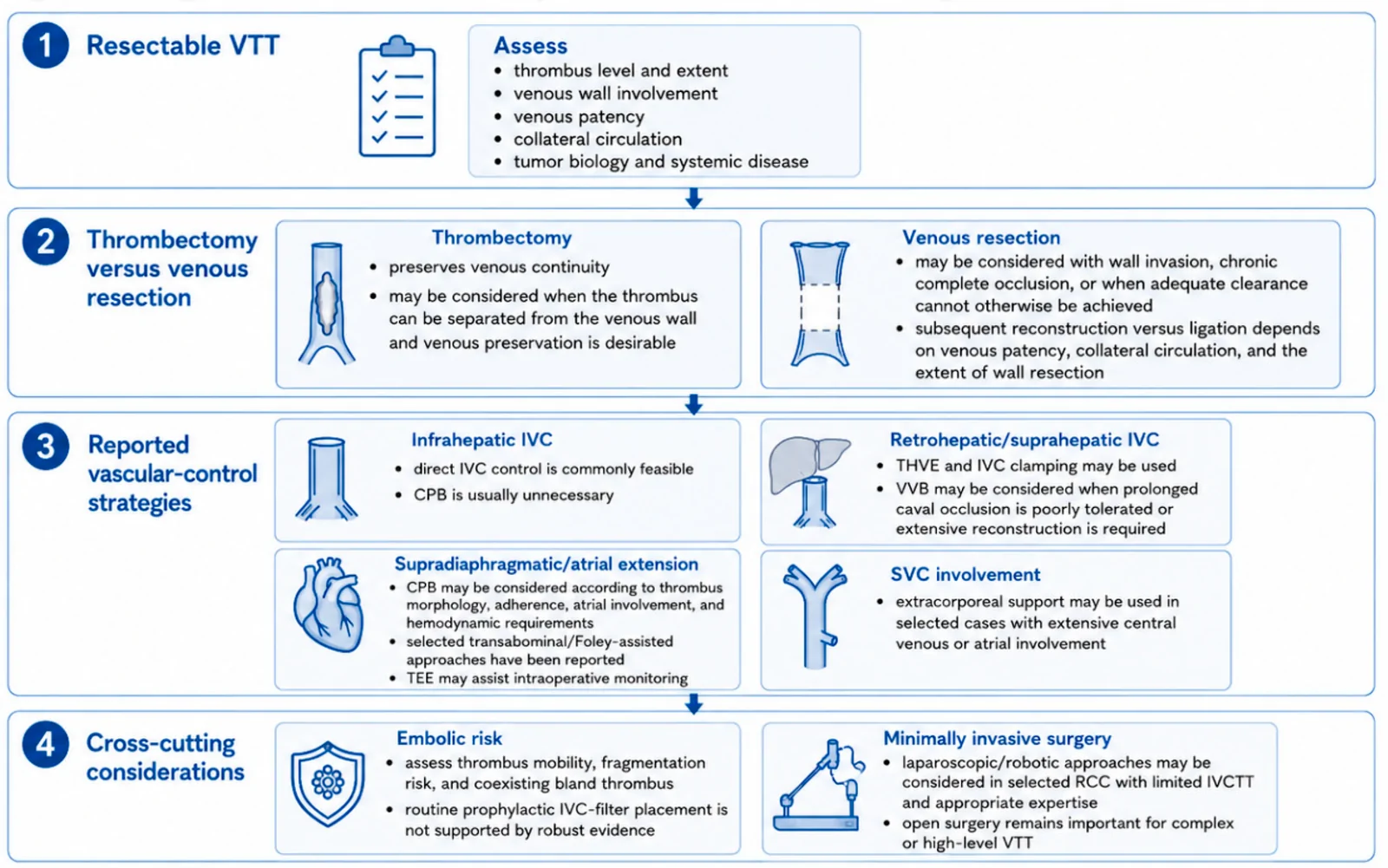
Surgical considerations and reported vascular-control strategies for venous tumor thrombus (VTT). The framework begins with assessment of resectable VTT according to thrombus level and extent, venous wall involvement, venous patency, collateral circulation, and the biology and systemic status of the underlying malignancy. The subsequent surgical strategy includes selection between thrombectomy and venous resection, followed by vascular-control techniques according to thrombus level and morphology. Cross-cutting considerations include embolic risk and the potential role of minimally invasive surgery. The approaches shown represent strategies reported in the literature and should be individualized according to tumor type, anatomy, hemodynamic considerations, and institutional expertise. Abbreviations: VTT, venous tumor thrombus; IVC, inferior vena cava; IVCTT, inferior vena cava tumor thrombus; SVC, superior vena cava; THVE, total hepatic vascular exclusion; VVB, venovenous bypass; CPB, cardiopulmonary bypass; TEE, transesophageal echocardiography; RCC, renal cell carcinoma.

For patients with resectable VTT, surgery remains an important treatment modality, particularly when complete removal of both the primary tumor and the intravascular tumor component is feasible [6,17,54,82]. The main objectives are to achieve oncological clearance; reduce the risk of thrombus-related complications, including pulmonary embolism [148]; maintain adequate venous return; and minimize perioperative risk. Because VTT varies substantially in location, extent, venous wall involvement, and collateral circulation, surgical management should be individualized according to tumor biology, vascular anatomy, systemic disease status, and the overall treatment strategy (Figure 2).

A key determinant of surgical strategy is the anatomical extent of VTT. Several tumor-specific classification systems have been developed to support operative planning. In RCC, systems such as the Mayo, Neves, and Ciancio classifications stratify tumor thrombus according to cranial extension within the IVC [34,114,149,150,151,152]. In HCC, the Japanese staging system and Cheng’s classification categorize PVTT and HVTT/IVCTT according to involvement of segmental branches, major veins, or central venous structures [51,52,78,153,154,155,156,157,158,159,160]. For rarer malignancies such as pelvic sarcoma, classification systems based on iliac vein and IVC involvement have also been proposed [6]. Although these systems are tumor-specific, they share a common principle: the extent of venous involvement is a key determinant of surgical complexity and resectability. Rather than applying a single classification system across tumor types, clinicians should focus on the anatomical information provided by these systems to guide individualized surgical planning.

Another central decision is whether to perform thrombectomy or en bloc venous resection. Thrombectomy preserves venous continuity by opening the venous wall, removing the intraluminal tumor thrombus, and repairing the vein. This approach may reduce postoperative venous complications but carries a risk of positive vascular margins when the thrombus adheres to or invades the venous wall [1,32,52,53,142,161,162]. In contrast, en bloc resection removes the involved venous segment together with the tumor thrombus and may provide better local clearance when the venous wall is involved, although it increases surgical complexity and may require ligation or vascular reconstruction [33,35,141,146,161]. The choice between these approaches depends on preoperative imaging, intraoperative findings, venous wall invasion, venous patency, and the expected consequences of venous interruption.

Experience from common VTT-associated tumors provides practical examples. In RCC, renal vein tumor thrombus can often be removed together with the kidney and renal vein, whereas IVCTT is commonly managed by thrombectomy unless venous wall invasion requires partial or segmental venous resection [2,17,18,19,20]. In HCC, the role of surgery is more selective and varies among regional treatment frameworks. Surgical series, particularly from East Asia, have generally included patients with preserved hepatic functional reserve, technically resectable disease, limited intrahepatic tumor burden, and no or limited extrahepatic disease; the extent of PVTT and the presence of portal hypertension are also important considerations [11,49,51,52,53,55,56]. These data are largely retrospective and subject to substantial selection bias, and the role assigned to surgery differs among Eastern and Western treatment frameworks.

When surgery is selected for HCC with PVTT, management also depends on the relationship between the thrombus and the planned hepatic resection. VTT confined within the resection field can often be removed en bloc with the primary tumor, whereas thrombus extending into the main portal trunk or contralateral portal vein may require portal vein thrombectomy in carefully selected patients [49,52,53,55]. HVTT/IVCTT requires additional consideration of the level of hepatic venous or caval involvement; reported approaches include thrombectomy, venous wall resection with reconstruction, and, for selected high-level thrombi, extracorporeal support [4,5,9,10,54,57,58,77]. Preservation of adequate hepatic venous outflow and suprahepatic caval patency is important to avoid severe postoperative outflow obstruction.

In osteosarcoma and other sarcomas, evidence remains limited. Complete excision of VTT within branch veins has generally been favored when feasible, whereas for thrombus involving major venous trunks, thrombectomy may be considered when the lumen remains partially patent, and en bloc resection may be more appropriate when the vein is chronically occluded or when wall invasion is suspected [6,12,100,101,102,103,104]. These approaches are based largely on small retrospective series and case reports and should not be regarded as established tumor-specific standards.

The need for venous reconstruction should also be assessed individually. Reconstruction is commonly considered after superior vena cava (SVC) resection when interruption of venous return is expected to result in clinically significant obstruction [83,90,163,164,165]. For IVC involvement, reconstruction may be considered when the lumen remains patent preoperatively, collateral circulation is inadequate, major collateral pathways are disrupted during surgery, or a substantial portion of the venous wall is resected [15,114,140,141,166]. In contrast, ligation or resection without reconstruction may be tolerated in selected patients with chronic complete occlusion and well-developed collateral circulation [141,146]. Careful preoperative evaluation of venous patency and collateral pathways is therefore important when planning venous resection.

Advanced vascular control techniques may be required in selected cases. The choice of technique depends on thrombus level and morphology, venous wall involvement, hemodynamic tolerance, tumor type, and institutional experience. Cardiopulmonary bypass (CPB) has been used for selected SVC tumor thrombi, particularly when there is extensive central venous or atrial involvement [163,164,165]. For IVCTT, thrombi below the hepatic veins can usually be managed without CPB using direct vascular control. For thrombi extending above the hepatic veins but remaining below the diaphragm, techniques such as total hepatic vascular exclusion (THVE) and IVC clamping have been used [141]. Venovenous bypass (VVB) may be considered when prolonged suprahepatic IVC occlusion is poorly tolerated or when more extensive venous wall resection or reconstruction is required [1,19,148].

For supradiaphragmatic IVCTT, CPB may be considered according to the extent and morphology of the thrombus, particularly when the intra-atrial component is bulky, the cranial end is wider than the caudal end, or the thrombus extends into the pulmonary vasculature or is adherent to the venous or atrial wall [1,10,19,141,167,168,169]. In carefully selected patients without venous wall or atrial invasion or adhesion, transabdominal approaches, including Foley catheter-assisted extraction, have been reported as alternatives to CPB, thoracotomy, or supradiaphragmatic IVC dissection [152,170,171]. In published series, patient selection for this technique has included respiratory mobility of the thrombus, favorable thrombus morphology, and limited intra-atrial extension; one series used a cranial thrombus diameter no greater than the caudal diameter and an intra-atrial extension of no more than 3 cm as selection criteria [171,172]. Potential risks include failed extraction, thrombus fragmentation with pulmonary embolism, residual VTT, and arrhythmia, making real-time intraoperative monitoring with TEE important [148,171,172].

The role of IVC filters in patients with VTT remains uncertain, and there is currently no consensus supporting routine prophylactic placement. Although filters have been used in selected patients considered to be at high risk of embolization, particularly when a bland thrombus component coexists, the available evidence is limited [148,173,174,175]. Filter placement may complicate proximal IVC control and surgical exposure and may be associated with penetration of the venous wall, thrombosis, incorporation into the tumor thrombus, or difficulties with subsequent retrieval. When temporary filter placement is considered, its position and timing should be planned in relation to the thrombus tip and the anticipated site of vascular clamping. Thus, filter use should be individualized according to thrombus morphology, associated bland thrombosis, anticipated embolic risk, and the planned surgical strategy [16,114,148,176]. The decision regarding filter retrieval can be difficult, requiring a balance between complications associated with prolonged filter placement and the potential risk of recurrent VTT with subsequent embolic events.

Minimally invasive surgery has expanded the treatment options for selected patients, particularly those with RCC and limited IVCTT. Laparoscopic and robotic approaches have been associated with lower blood loss, shorter hospital stay, and favorable perioperative outcomes in experienced centers [166,168,177,178,179,180,181,182,183,184,185,186]. A recent systematic review and network meta-analysis of 66 studies including 3241 patients with nonmetastatic RCC and IVCTT found lower blood loss, fewer overall complications, and shorter hospital stay with minimally invasive approaches compared with open surgery. However, most included studies were retrospective, and minimally invasive surgery was preferentially used in selected patients, mainly those with Mayo level I–II thrombi; major complication rates were not significantly different in the network analysis, and oncological outcomes were incompletely reported. These findings therefore do not establish superiority of minimally invasive surgery over open surgery [187]. Open surgery remains an important approach for complex or high-level VTT, while laparoscopic and robotic approaches may be appropriate for selected patients in centers with relevant expertise.

## 6. Non-Surgical Management of Venous Tumor Thrombus

Non-surgical treatments are important components of multidisciplinary VTT management. They may be used before surgery to reduce tumor burden, after surgery to reduce recurrence risk, as definitive treatment for unresectable disease, or for disease control in patients who are not surgical candidates. Their role varies substantially according to tumor type, thrombus extent, tumor biology, resectability, and sensitivity to systemic or locoregional treatment.

In RCC, earlier experience with targeted agents such as sunitinib and sorafenib showed variable and generally modest effects on VTT. In a multi-institutional series of 25 patients treated with targeted therapy, the thrombus level decreased in only a minority of patients, and a subsequent 14-patient series similarly found limited effects on thrombus level and surgical planning [21,22,114]. Later retrospective studies suggested that selected patients may achieve greater thrombus reduction; for example, a multicenter study of neoadjuvant sunitinib reported a decrease in IVC thrombus level in 8 of 19 treated patients [23].

Prospective and more recent studies have provided further evidence for presurgical systemic therapy. In the single-arm phase II NAXIVA trial, 20 patients with resectable clear-cell RCC and VTT received presurgical axitinib; the Mayo thrombus level decreased in 7 patients (35%), and 7 of 17 patients who underwent surgery had a less invasive operation than initially planned [24]. Small series subsequently reported VTT responses to immune checkpoint inhibitor (ICI)-based therapy and ICI/tyrosine kinase inhibitor (TKI) combinations [25,26]. In the single-arm phase II NEOTAX study, 25 patients with clear-cell RCC and IVCTT received toripalimab plus axitinib; the thrombus level decreased in 11 patients (44%) and the planned surgical strategy was changed in 13 of 21 patients who underwent surgery, although three experienced major surgical complications [27].

The overall evidence remains heterogeneous. A 2024 systematic review and meta-analysis including 204 patients treated with neoadjuvant therapy reported a pooled thrombus-level reduction rate of 29.4%; neoadjuvant treatment was associated with shorter operative time and less blood loss, but complication rates and oncological outcomes were not significantly different from those with upfront surgery [28]. More recent retrospective studies have also reported thrombus responses to ICI-based or ICI/TKI treatment, although reduction in thrombus size does not always translate into a lower anatomical thrombus level [29,30,31]. Taken together, presurgical systemic therapy can reduce VTT burden and may simplify surgery in a subset of patients, but the available evidence does not yet establish routine neoadjuvant therapy as a standard approach for resectable RCC with VTT. After complete resection, adjuvant pembrolizumab has demonstrated disease-free and overall survival benefit in selected patients with clear-cell RCC at increased risk of recurrence [188]. However, this evidence is based on postoperative recurrence risk in clear-cell RCC rather than on VTT-specific trials, and the presence or level of VTT itself has not been established as a predictive marker for adjuvant systemic therapy.

In HCC, non-surgical treatment has a central role, particularly for patients with PVTT, HVTT or IVCTT who are not suitable for surgery. Modern systemic therapy, increasingly based on ICI-containing regimens, has changed the treatment of unresectable HCC with macrovascular invasion. In an exploratory analysis of the IMbrave150 trial, atezolizumab plus bevacizumab was evaluated in patients with Vp4 PVTT involving the main portal trunk and/or contralateral portal vein; median overall survival was 7.6 months in 48 patients treated with atezolizumab plus bevacizumab and 5.5 months in 25 patients treated with sorafenib [60]. These subgroup data support the activity of ICI-based systemic therapy in advanced PVTT, although they are not derived from a trial designed specifically for this population.

Locoregional therapies remain relevant in selected patients with HCC-associated VTT. Radiotherapy has been used for local control of PVTT and other forms of macrovascular invasion [59,61,62]. The role of transarterial chemoembolization (TACE) depends on PVTT extent, hepatic functional reserve, residual portal flow, intrahepatic tumor burden, and the risk of hepatic decompensation; it should therefore not be regarded as a uniform option for all patients with PVTT [9,10,11,51,63,64,65,66]. Combinations of TACE and radiotherapy or systemic treatment have shown encouraging outcomes in selected populations [67,68,69,70,71]. More recent studies have also explored combinations of radiotherapy or other locoregional therapies with TKI and ICI treatment in HCC with PVTT, with promising results, although much of the evidence remains retrospective and prospective confirmation is needed [72,73].

Transarterial radioembolization (TARE) with yttrium-90 microspheres has also been investigated in selected patients with HCC and PVTT [74], but its use and expected benefit depend on patient selection and institutional expertise. Portal vein stenting has been used in selected patients with main PVTT to restore portal flow or facilitate subsequent locoregional treatment [75,76]; however, evidence remains limited and this approach is not broadly established as standard treatment.

For sarcomas, the effect of non-surgical treatment on VTT is less predictable and is strongly influenced by histologic subtype. Chemotherapy and radiotherapy may be effective in chemosensitive and radiosensitive tumors such as Ewing sarcoma, and complete response of VTT after combined chemotherapy and radiotherapy has been reported [105]. In contrast, osteosarcoma-associated VTT appears less responsive to chemotherapy, and the role of radiotherapy remains uncertain [6,12,100,101,102,103,104]. Because the available evidence consists largely of small retrospective series and case reports, non-surgical treatment of sarcoma-associated VTT should primarily follow the treatment sensitivity and clinical context of the underlying sarcoma.

Anticoagulation should be distinguished from antitumor treatment of VTT. Because VTT consists predominantly of malignant tissue rather than fibrin-rich thrombus, anticoagulation does not eliminate the tumor component, and current evidence does not support routine anticoagulation solely because VTT is present. A recent systematic review of six observational studies involving 659 patients with RCC-associated tumor thrombus found that anticoagulation may reduce venous thromboembolic events in some patients, but bleeding was also frequent and the available evidence remained inconclusive [130]. Similarly, a cohort study of RCC-associated VTT found a substantially increased risk of venous thromboembolism; patients receiving anticoagulation continued to experience thromboembolic events, with a higher observed risk of major bleeding [131].

Anticoagulation may nevertheless be appropriate when VTT coexists with bland thrombus or when there is another conventional indication, such as deep-vein thrombosis, pulmonary embolism, or catheter-associated thrombosis. In these situations, the expected thrombotic benefit should be balanced against bleeding risk. This consideration is particularly important in HCC, where portal hypertension and gastroesophageal varices may increase the risk of hemorrhage. In a retrospective study of 124 patients with HCC and macrovascular tumor invasion, 40% had an additional non-tumorous thrombus component; anticoagulation was not associated with a significant change in malignant thrombosis, whereas appropriate management of varices was associated with a lower risk of variceal bleeding [132]. Thus, anticoagulation decisions should be based on the presence of a non-tumorous thrombotic component or another thromboembolic indication and on the individual balance between thrombosis and bleeding, rather than on VTT alone [133].

Overall, non-surgical treatment of VTT should be determined primarily by the underlying malignancy, systemic disease status, thrombus anatomy, resectability, and treatment sensitivity. In RCC, presurgical systemic therapy is increasingly being investigated as a means of reducing VTT burden and surgical complexity, but its routine role remains uncertain. In HCC, systemic and locoregional therapies are increasingly integrated according to PVTT extent, hepatic reserve, tumor burden, and extrahepatic disease. Evidence for rarer malignancies remains limited.

## 7. Prognosis of Venous Tumor Thrombus

VTT is frequently associated with adverse oncological outcomes, although its prognostic significance varies substantially according to tumor type, thrombus extent, metastatic status, treatment strategy, and the feasibility of complete tumor clearance [6,7,8]. Therefore, survival outcomes reported for different malignancies or treatment approaches should be interpreted within their specific clinical context rather than compared directly across studies.

RCC is one of the best-studied VTT-associated malignancies. In a historical cohort of untreated RCC with VTT, median survival was 5 months and 1-year disease-specific survival was 29% [8]. In contrast, selected surgical series have reported 5-year overall survival (OS) rates of approximately 32–54% and 10-year OS rates of 21–46% after radical nephrectomy and tumor thrombectomy [14,15,32,33,34,35,36,37,38,39,40,41,42,43,44,45]. These estimates are derived from heterogeneous retrospective cohorts and should not be interpreted as pooled survival rates. Adverse prognostic factors include distant metastasis, perirenal fat invasion, lymph node metastasis, and sarcomatoid differentiation [14,32,36,37,38,40,41,45]. Although IVCTT may be associated with poorer outcomes than renal vein tumor thrombus (RVTT), the cranial level of IVCTT itself does not consistently predict oncological outcome [14,17,35,38,39,45,46,47,48]. Thus, the presence of VTT in RCC does not necessarily preclude long-term survival, and prognosis appears to depend substantially on systemic disease status and tumor biology rather than thrombus level alone.

HCC represents a biologically and clinically different setting. In a historical untreated cohort, median survival was reported to be 24.4 months in patients without PVTT and 2.7 months in those with PVTT [7]. However, outcomes in HCC with VTT vary widely according to hepatic functional reserve, intrahepatic tumor burden, extrahepatic disease, vascular anatomy, and treatment strategy. Surgical resection has been associated with prolonged survival in selected patients [10,11,51,53,54,55], while systemic therapy, radiotherapy, TACE, TARE, and other locoregional approaches may be used according to the clinical setting [4,11,51,57,59,65,66,68,71,74,76,189]. PVTT involving the main portal trunk generally carries a poorer prognosis than branch-limited PVTT [49,50,55,64,65,67,71,75,78], and HVTT/IVCTT is also associated with poor outcomes, particularly when there is central venous or atrial extension [4,5,9,10,54,58,77,79]. These differences support considering HCC-associated VTT as a spectrum of vascular involvement rather than a single prognostic entity.

The prognostic significance of VTT also varies considerably among other malignancies. Wilms tumor with IVCTT or atrial tumor thrombus can achieve favorable outcomes with chemotherapy and surgery, with reported 3-year recurrence-free survival of 76.9% and 5-year OS of 82.2% [80,81,82]. In contrast, adrenocortical carcinoma with IVC or atrial extension is generally associated with advanced disease and poor prognosis [96,97,98,99]. VTT has also been reported in thyroid, testicular, gastric, and colorectal cancers, but the available evidence is limited, and its clinical impact depends largely on the biology of the underlying malignancy, the anatomical extent of vascular involvement, and the feasibility of local and systemic treatment [83,84,85,86,87,88,89,90,91,92,93,94,95].

Evidence regarding VTT in mesenchymal tumors is particularly limited [3,6,105,107,108,109,110,111,112]. In a retrospective single-institution series of pelvic bone sarcomas, macroscopic VTT in osteosarcoma was associated with lung metastasis at presentation and poorer OS, recurrence-free survival, and metastasis-free survival in patients with localized surgically treated disease [6]. In the same series, VTT resection was associated with better local control and longer OS or recurrence-free survival in selected patients but did not appear to substantially improve metastasis-free survival, suggesting that systemic progression remained an important determinant of outcome [6]. Other published evidence for sarcoma-associated VTT consists largely of small series and case reports. Thus, the reported epidemiological and prognostic associations require independent validation and may also reflect institution-specific patient selection and treatment practices.

Overall, the prognostic implications of VTT should be interpreted in the context of the underlying tumor biology, vascular territory, and systemic disease burden.

## 8. Transferable and Tumor-Specific Principles of VTT Management

Despite marked differences among malignancies, VTT management can be organized around several shared clinical questions: (1) does the intravascular lesion represent VTT, bland thrombus, or another venous mass; (2) what is the anatomical extent of VTT, including venous wall involvement, patency, and collateral circulation; (3) is complete resection technically feasible and oncologically appropriate; (4) what vascular-control or venous-reconstruction strategy is required; and (5) how should surgery be integrated with tumor-specific systemic or locoregional treatment? The first two questions are summarized in the imaging framework shown in Figure 1, while the surgical considerations are summarized in Figure 2. The final treatment decision, however, remains dependent on the biology, treatment sensitivity, organ function, and systemic extent of the underlying malignancy.

In contrast, the clinical significance of VTT and the choice of treatment cannot be directly extrapolated from one malignancy to another. In RCC, complete removal of localized disease together with the venous thrombus may provide long-term disease control in selected patients, and thrombus level often has greater implications for surgical complexity than for prognosis [14,17,35,38,39,45,46,47,48]. HCC-associated PVTT differs substantially because portal venous invasion is closely related to intrahepatic tumor burden, hepatic functional reserve, portal hypertension, and the extent of portal involvement; consequently, the roles of surgery, systemic therapy, and locoregional treatment depend on a different set of clinical considerations [10,11,51,53,54,55]. In sarcoma, treatment response also varies markedly by histologic subtype. VTT in Ewing sarcoma may respond to chemotherapy and radiotherapy, whereas osteosarcoma-associated VTT appears less responsive to chemotherapy and may accompany a high risk of metastatic progression [6]. Thus, similar anatomical patterns of venous extension do not necessarily imply similar tumor biology, prognosis, or treatment response.

For rarer malignancies, experience from RCC and HCC may therefore be most useful in defining the questions that should be addressed rather than determining the treatment itself. Anatomical assessment and vascular surgical principles may be informative across tumor types, but the indication for surgery, the role of systemic or locoregional therapy, and the expected oncological benefit should remain guided by the biology and treatment sensitivity of the underlying malignancy. This distinction is particularly important because the available evidence is uneven and is largely retrospective, while data for rare VTT-associated tumors often come from small series or case reports. Cross-tumor principles should therefore be viewed as a framework for clinical assessment and multidisciplinary discussion rather than as uniform treatment recommendations.

## 9. Conclusion and Future Perspectives

VTT remains a challenging manifestation of malignancy for which the evidence base is highly uneven. Available data are strongest in RCC and HCC, whereas evidence for rarer malignancies, particularly sarcomas, is limited and often derived from retrospective single-institution series or case reports.

Future studies should focus on standardized reporting of VTT anatomy, treatment, and outcomes and on multicenter collaboration for rare malignancies. Prospective evaluation of emerging systemic and locoregional therapies, particularly their integration with surgery, will be important for refining patient selection and determining whether improved control of VTT translates into better oncological outcomes.

## Figures and Tables

**Table 1:** Clinical characteristics of venous tumor thrombus across major malignancies.

Malignancy	Typical Venous Involvement and Reported Incidence	Surgical Considerations	Systemic/Locoregional Treatment	Prognostic Implications	Evidence Context
Renal cell carcinoma (RCC)	Renal vein and IVC involvement. Venous extension involving the renal vein and/or IVC has been reported in >20% of cases; IVCTT occurs in approximately 4–10% [1,2,13,14,15,16].	Renal vein thrombus can often be removed with the kidney and renal vein. IVCTT is commonly managed by thrombectomy; partial or segmental venous resection may be required with wall invasion [2,17,18,19,20].	Earlier TKI-based therapy showed variable effects on VTT. Presurgical TKI or ICI/TKI therapy can reduce thrombus burden or level in a subset of patients, but routine neoadjuvant treatment remains investigational [21,22,23,24,25,26,27,28,29,30,31].	Selected surgical series report long-term survival after complete resection. Prognosis is strongly influenced by metastatic status and tumor biology, while IVCTT level alone does not consistently predict survival [8,14,15,32,33,34,35,36,37,38,39,40,41,42,43,44,45,46,47,48].	Most extensively studied VTT-associated malignancy; evidence remains predominantly retrospective, with several recent prospective phase II studies of presurgical therapy.
Hepatocellular carcinoma (HCC)	Most commonly PVTT (approximately 8.9–46% across different clinical series); HVTT/IVCTT occurs in approximately 1.4–4.9% [5,9,11,49,50].	Resection is considered in selected patients and varies among regional treatment frameworks. Surgical selection depends on hepatic reserve, tumor burden, PVTT extent, portal hypertension, extrahepatic disease, and technical resectability [11,49,51,52,53,54,55,56,57,58].	ICI-based systemic therapy has an increasing role in unresectable disease. RT, TACE, TARE, systemic therapy, and selected interventional approaches may be used alone or in combination according to PVTT extent and hepatic reserve [59,60,61,62,63,64,65,66,67,68,69,70,71,72,73,74,75,76].	VTT is generally an adverse prognostic feature. Main portal-trunk PVTT carries poorer prognosis than branch-limited PVTT; central HVTT/IVCTT is also associated with poor outcomes [4,5,7,9,10,49,50,54,55,58,64,65,67,71,75,77,78,79].	Extensive literature, but substantial heterogeneity in liver function, VTT classification, treatment strategy, and geographic practice.
Wilms tumor	IVC or atrial tumor thrombus in approximately 6–11.3% [80,81,82].	Surgery is integrated with chemotherapy and depends on the cranial extent of the thrombus [80,81,82].	Chemotherapy is an important component of treatment and may facilitate subsequent resection [80,81,82].	Relatively favorable outcomes have been reported, including 5-year OS of approximately 82% [80,81,82].	Evidence is more limited than RCC/HCC but includes relatively large pediatric cooperative-group series.
Other malignancies	IVCTT has been reported in testicular tumors (~0.6–2.5%) and VTT in colorectal cancer (~1–2%); macroscopic VTT is rare in gastric and thyroid malignancies [83,84,85,86,87,88,89].	Management depends strongly on the underlying malignancy and anatomical extent [83,84,85,86,87,88,89,90,91,92,93,94,95].	Treatment follows the biological and therapeutic characteristics of the primary malignancy.	Clinical impact is highly variable among tumor types [83,84,85,86,87,88,89,91,92,93,94,95,96,97,98,99].	Evidence is heterogeneous and frequently limited to small series and case reports.
Sarcomas	In a single-institution series of pelvic bone sarcomas, macroscopic VTT occurred in 22.6% of osteosarcomas, 3.8% of chondrosarcomas, 1.4% of Ewing sarcomas, and 3.8% of undifferentiated pleomorphic sarcomas [3,6].	Complete excision is pursued when feasible. Thrombectomy versus venous resection is selected according to venous patency, wall involvement, anatomical extent, and collateral circulation [6,12,100,101,102,103,104].	Response is histology-dependent. Ewing sarcoma VTT may respond to chemotherapy and RT, whereas osteosarcoma-associated VTT appears less responsive to chemotherapy [6,100,101,102,103,104,105].	Osteosarcoma-associated VTT has been associated with metastatic presentation and poorer outcomes; systemic progression remains an important determinant of prognosis [3,6].	Cohort-level evidence is scarce; several of the larger published series originate from a single institution, while much of the remaining literature consists of case reports.

**Table 2:** Imaging features useful in differentiating venous tumor thrombus, bland thrombus, and primary venous sarcoma.

Feature	Venous Tumor Thrombus	Bland Thrombus	Primary Venous Sarcoma
Relationship to primary tumor	Usually contiguous with an identifiable primary tumor or extends along adjacent venous structures [3,50,77,113].	No direct continuity with the primary tumor.	Arises from the venous wall; an organ-based primary tumor is usually absent [119,120,121].
Contrast enhancement	Internal enhancement favors VTT; enhancement may resemble that of the primary tumor [3,6,116,117].	Usually lacks internal enhancement [118].	Heterogeneous internal enhancement may occur [119,120,121,122].
Venous morphology	Luminal expansion may occur [3,6].	Variable according to thrombus age and extent.	Wall-centered, eccentric or circumferential growth; extraluminal extension may occur [119,120,121].
MRI characteristics	Signal characteristics may resemble the primary tumor; diffusion restriction may occur [3,6,116,117].	Signal may evolve over time [118].	Tumor-like signal and diffusion restriction may occur [120,121,122].
^18^F-FDG PET/CT	Increased uptake may support VTT in FDG-avid malignancies [123,124,125,126].	Usually lacks tumor-like metabolic activity.	Increased metabolic activity may occur [120,121,122].
Additional imaging clues	Continuity with the primary tumor; calcification or ossification may occur in osteosarcoma [3,6]. In HCC, enhancement and expansion of the portal vein support PVTT [127].	Temporal evolution may support bland thrombus; bland thrombus may coexist with VTT.	Wall-centered origin and a substantial extraluminal component favor primary venous tumor.
Useful modalities	Contrast-enhanced CT and MRI; US, CEUS, and ^18^F-FDG PET/CT in selected settings [114,115,116,117,118,123,124,125,126,128].	Contrast-enhanced CT/MRI; US and CEUS as appropriate [118,128].	CT and MRI; PET/CT and transvenous biopsy in selected indeterminate lesions [119,120,121,122,129].
Important limitations	Appearance varies according to tumor type, venous territory, and imaging protocol.	May coexist with VTT, resulting in different characteristics within the same venous segment.	Rare; imaging and biopsy evidence is derived mainly from small series.
Clinical implication	Represents malignant intravascular extension and influences oncological and surgical planning.	May provide a separate indication for anticoagulation depending on thrombotic and bleeding risks [130,131,132,133].	Represents a separate primary malignancy requiring tumor-specific management [119,120,121].

Note: No single imaging feature reliably distinguishes these entities in all settings. Imaging findings should be interpreted according to the primary tumor, venous territory, temporal evolution, and clinical context. Abbreviations: VTT, venous tumor thrombus; RCC, renal cell carcinoma; HCC, hepatocellular carcinoma; IVC, inferior vena cava; IVCTT, inferior vena cava tumor thrombus; PVTT, portal vein tumor thrombus; HVTT, hepatic vein tumor thrombus; TKI, tyrosine kinase inhibitor; ICI, immune checkpoint inhibitor; RT, radiotherapy; TACE, transarterial chemoembolization; TARE, transarterial radioembolization; OS, overall survival; CT, computed tomography; MRI, magnetic resonance imaging; US, ultrasonography; CEUS, contrast-enhanced ultrasound; FDG, fluorodeoxyglucose; PET/CT, positron emission tomography/computed tomography.

## Data Availability

No new data were created or analyzed in this study. Data sharing is not applicable to this article.

## Abbreviations

CEUS	Contrast-enhanced ultrasound
CPB	Cardiopulmonary bypass
CT	Computed tomography
FDG	Fluorodeoxyglucose
HCC	Hepatocellular carcinoma
HVTT	Hepatic vein tumor thrombus
ICI	Immune checkpoint inhibitor
IVC	Inferior vena cava
IVCTT	Inferior vena cava tumor thrombus
MRI	Magnetic resonance imaging
OS	Overall survival
PET/CT	Positron emission tomography/computed tomography
PVTT	Portal vein tumor thrombus
RCC	Renal cell carcinoma
RT	Radiotherapy
RVTT	Renal vein tumor thrombus
SVC	Superior vena cava
TACE	Transarterial chemoembolization
TARE	Transarterial radioembolization
TEE	Transesophageal echocardiography
THVE	Total hepatic vascular exclusion
TKI	Tyrosine kinase inhibitor
US	Ultrasonography
VTT	Venous tumor thrombus
VVB	Venovenous bypass
